# Supplementary material for: Multiple p38/JNK mitogen-activated protein kinase (MAPK) signaling pathways mediate salt chemotaxis learning in C. elegans
Source: G3 (Bethesda). 2023 Jun 13;13(9):jkad129. doi: 10.1093/g3journal/jkad129 (PMC10468299; doi:10.1093/g3journal/jkad129)
Supplement: jkad129_Supplementary_Data [file jkad129_supplementary_data.zip › Supplemental_Material_G3-2023-404264.pdf]

Supplementary Figure S1

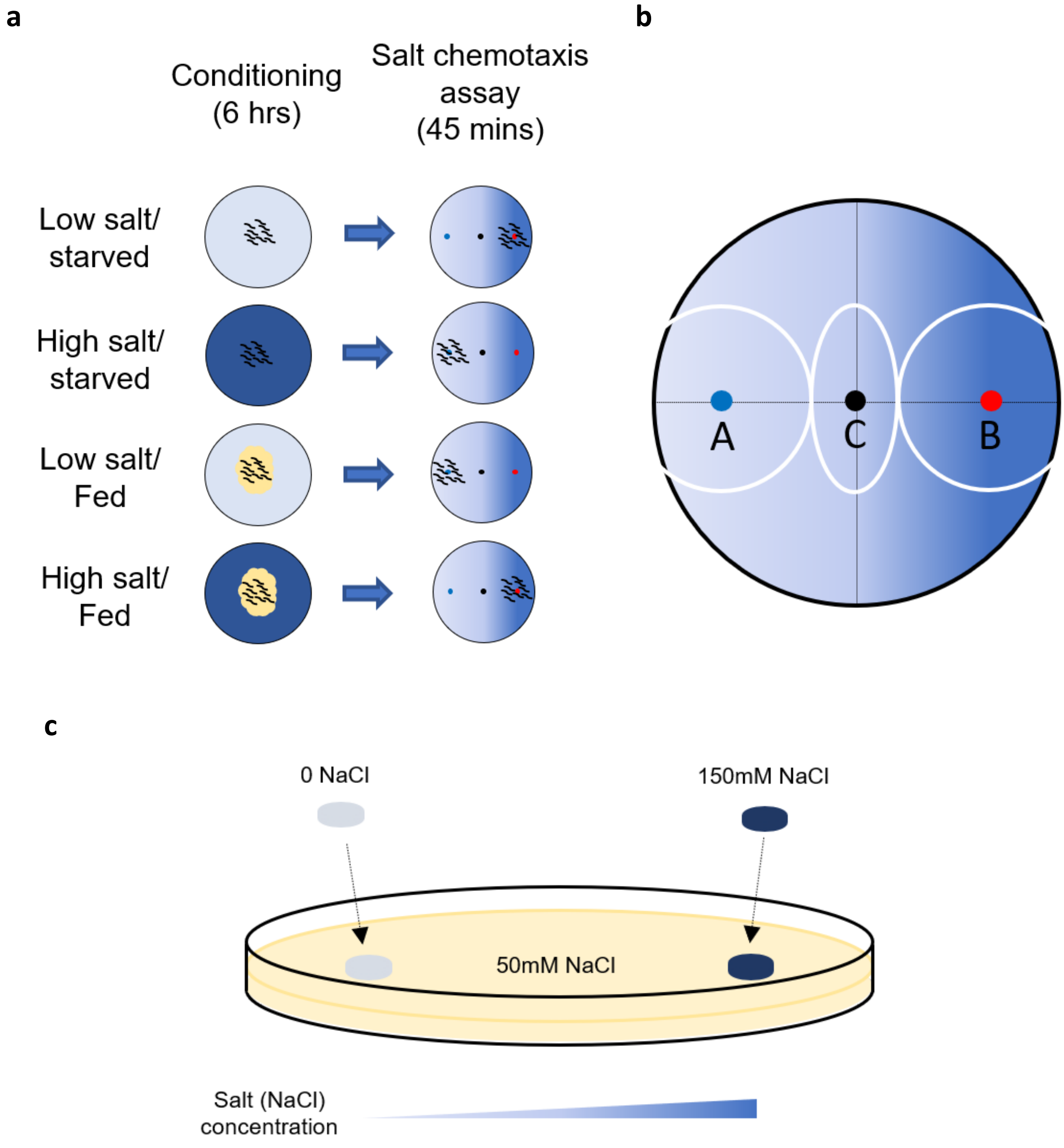

Supplementary Figure S2

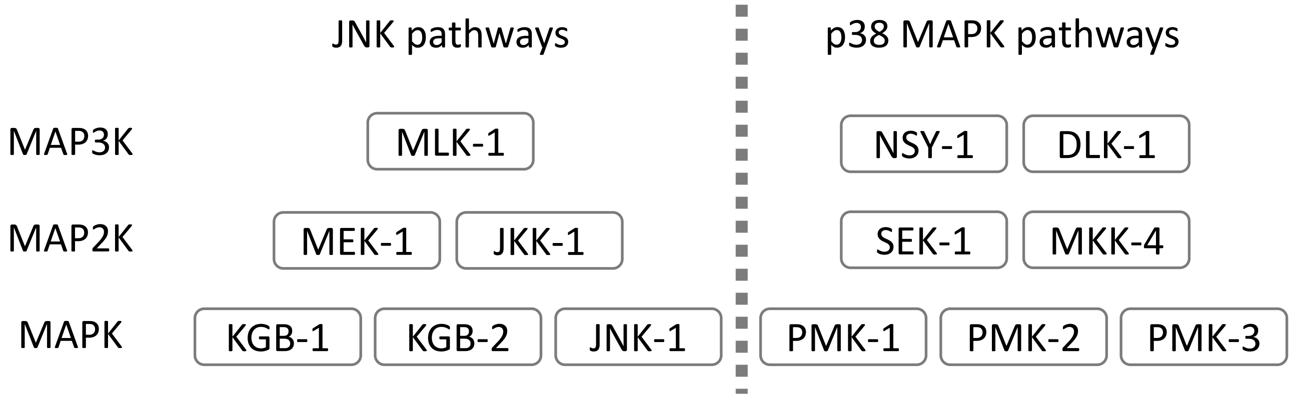

Supplementary Figure S3

a

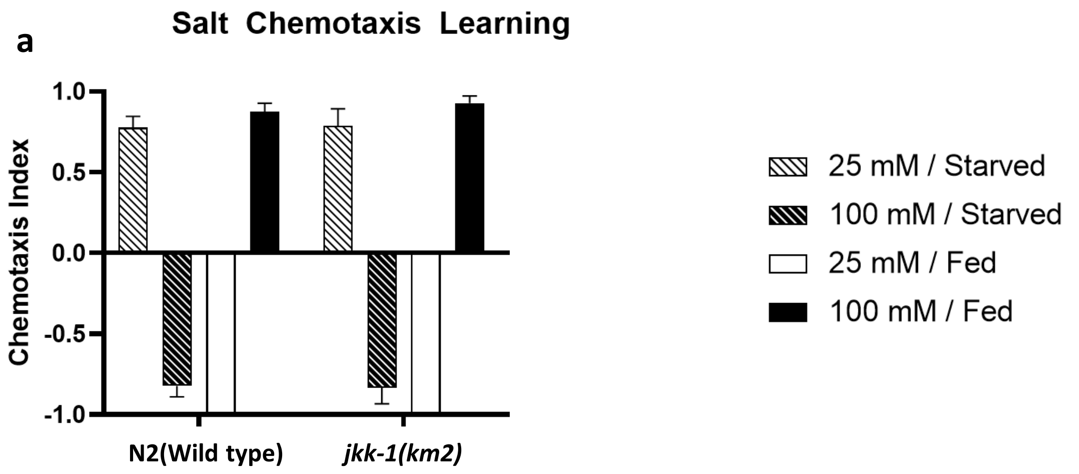

b

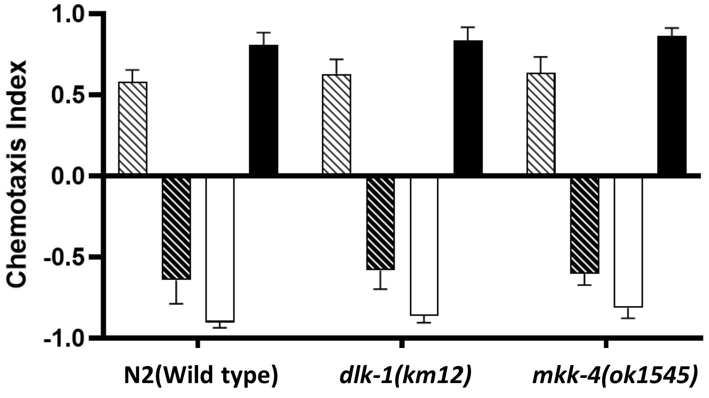

Supplementary Figure S4

a

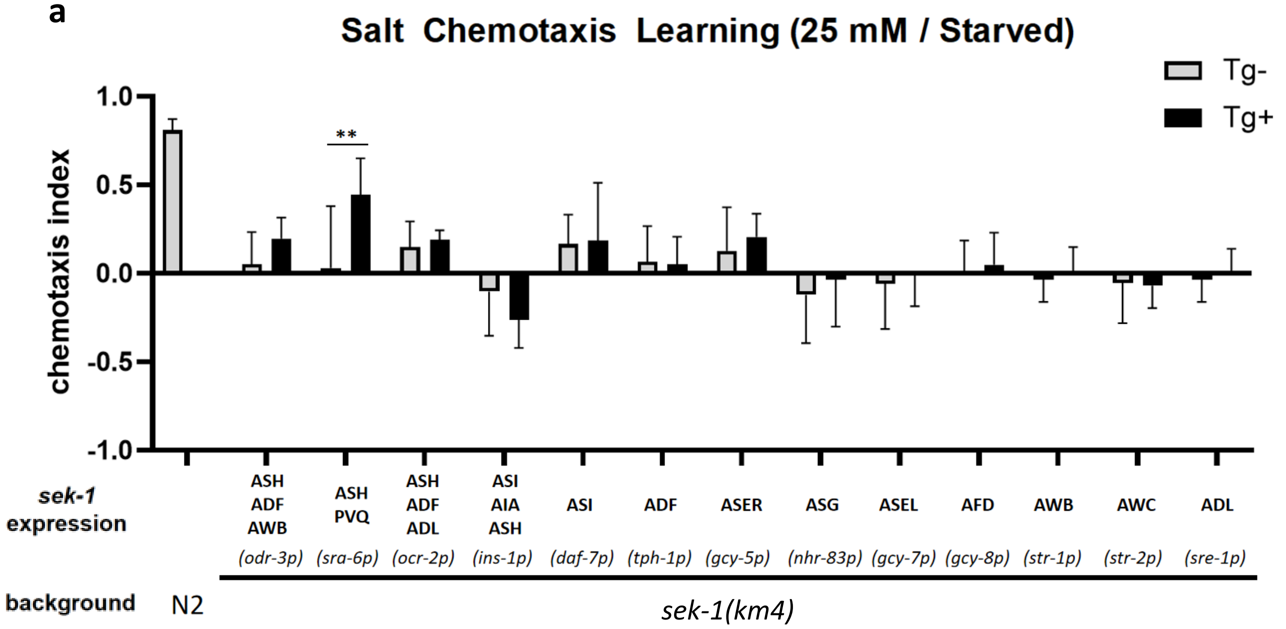

b

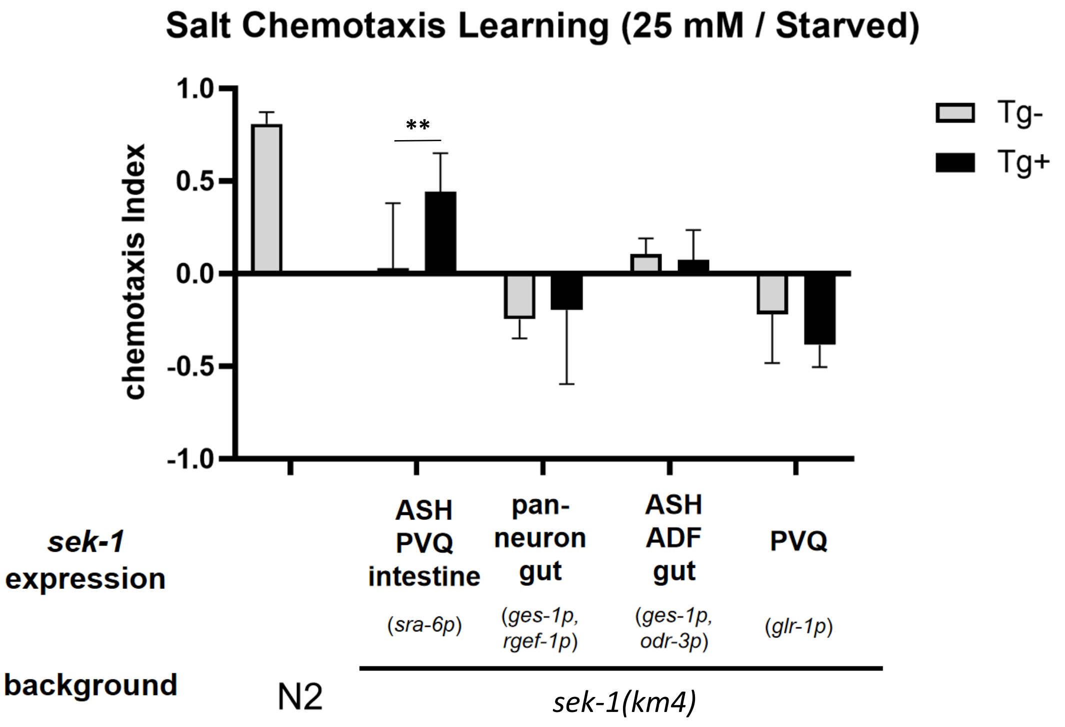

Supplementary Figure S5

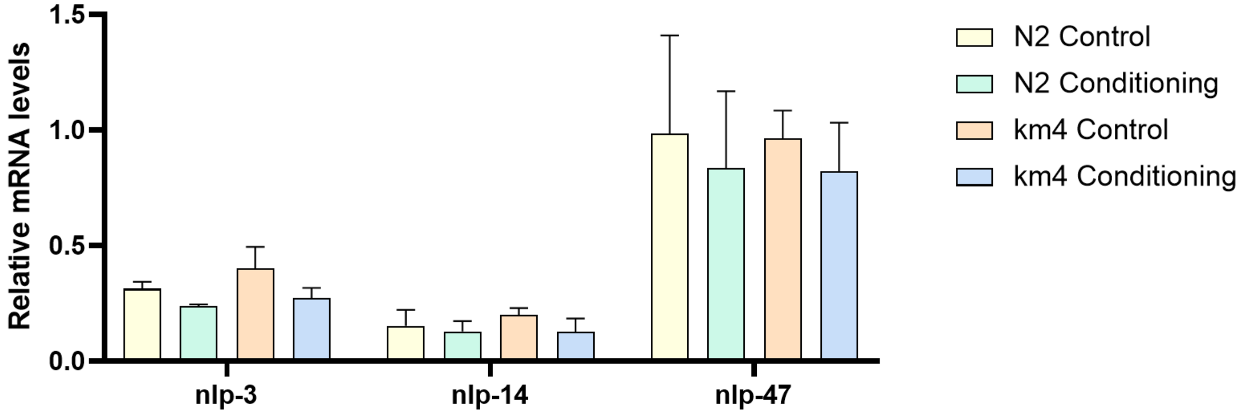

Supplementary Figure S6

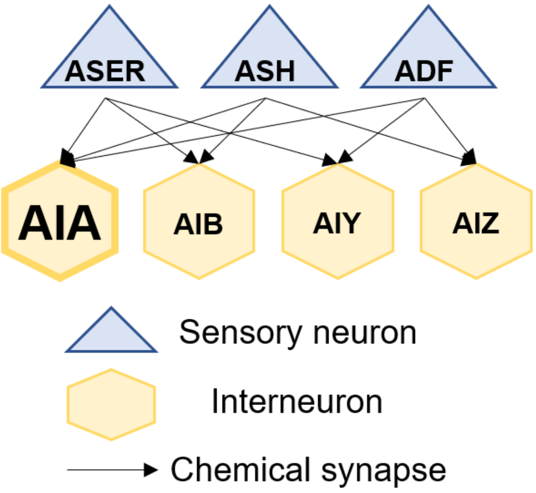

Supplementary Figure S7

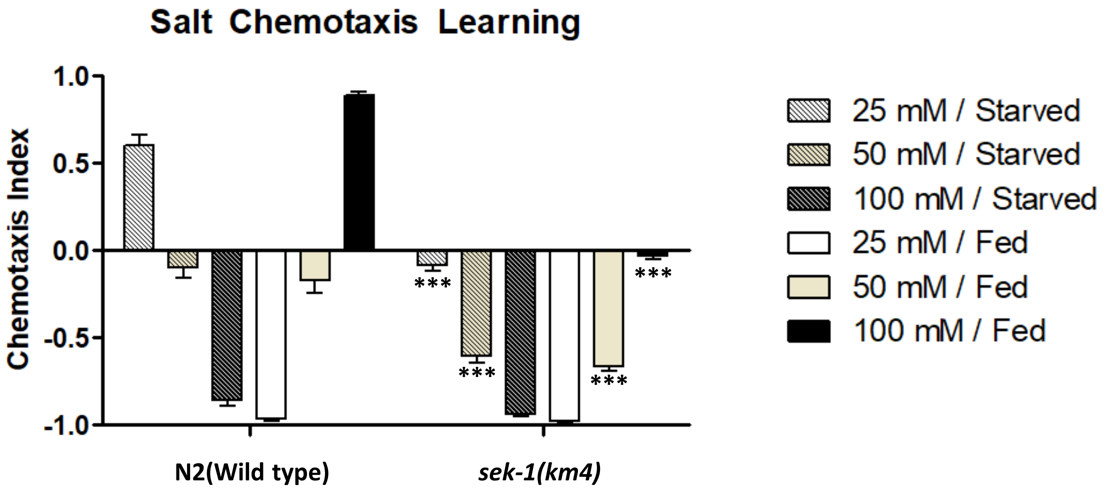

Supplementary Figure S8

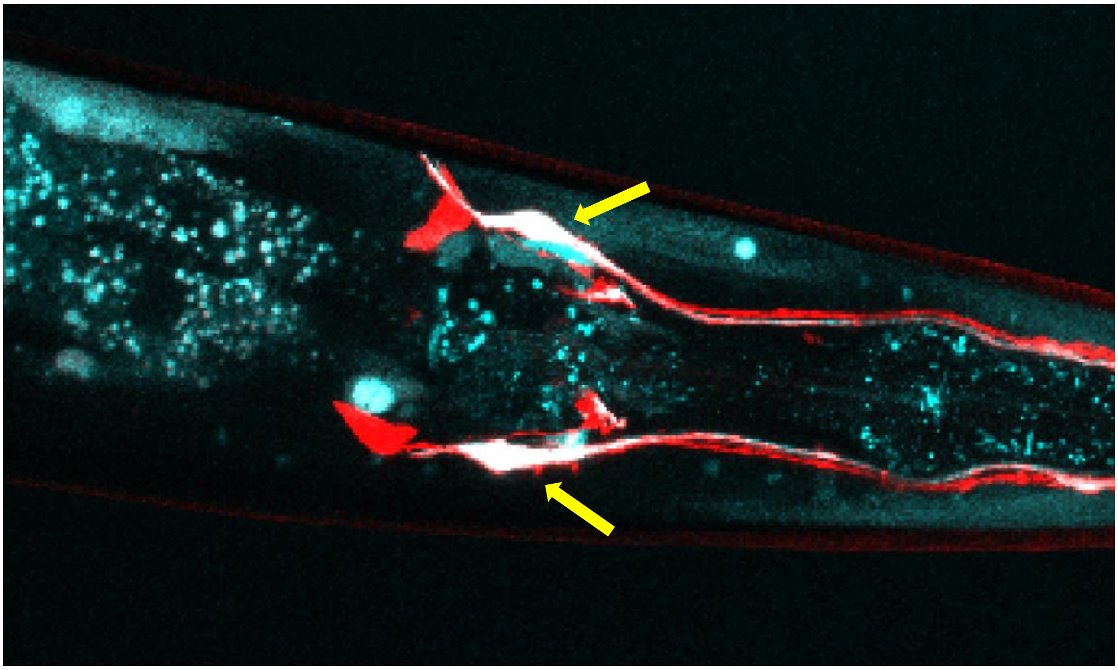

Supplementary Table S1. Strains and transgenic lines

Strain	Genotype	Type
N2	Wild-type	Reference strain
KU4	*sek-1(km4) X.*	Mutant Strain
AU1	*sek-1(ag1) X.*	
CB408	*unc-43(e408) IV.*	
IG685	*tir-1(tm3036) III.*	
AU3	*nsy-1(ag3) II.*	
KU25	*pmk-1(km25) IV.*	
JN5707	*pmk-2(pe5707)IV.*	
BS3383	*pmk-3(ok169) IV*	
VC513	*grk-2(gk268) III.*	
HA865	*grk-2(rt97) III.*	
RB660	*arr-1(ok401) X.*	
JN3745	*mek-1(pe3745) X.*	
RB1908	*mlk-1(ok2471) V.*	
KU12	*dlk-1(km12) I.*	
KU23	*mkk-4(km23) X.*	
KU2	*jkk-1(km2) X.*	
VC8	*jnk-1(gk7) IV.*	
KB3	*kgb-1(um3) IV.*	
VC822	*kgb-2(gk361) IV.*	
FX03023	*nlp-3(tm3023) X.*	
FX01880	*nlp-14 (tm1880) X.*	
	nlp-47(tm13464) IV.	
RB1911	*ins-27(ok2427) I.*	
	npr-4 (tm1782) X.	
	npr-12 (tm1498) IV.	
FX02048	*npr-15 (tm2048) III.*	
RB1405	*npr-22(ok1598) IV.*	
JN5708	*sek-1(km4) X.; peEx5708[rgef-1p::sek-1::sl2::cfp(5ng); myo-3p::venus(10ng)]*	Transgenic Lines
JN5709	*sek-1(km4) X.; peEx5709[odr-3p::sek-1::sl2::cfp(5ng); myo-3p::venus(10ng)]*	
JN5711	*sek-1(km4) X.; peEx5711[sra-6p::sek-1::sl2::cfp(5ng); myo-3p::venus(10ng)]*	
JN5712	*sek-1(km4) X.; peEx5712[ocr-2p::sek-1::sl2::cfp(5ng); myo-3p::venus(10ng)]*	
JN5714	*sek-1(km4) X.; peEx5714[gcy-5p::sek-1::sl2::cfp(5ng); myo-3p::venus(10ng)]*	
JN5715	*sek-1(km4) X.; peEx5715[gcy-7p::sek-1::sl2::cfp(5ng); myo-3p::venus(10ng)]*	

Strain	Genotype	Type
JN5716	*sek-1(km4) X.; peEx5716[gcy-8p::sek-1::sl2::cfp(5ng); myo-3p::venus(10ng)]*	Transgenic Lines
JN5717	*sek-1(km4) X.; peEx5717[str-1p::sek-1::sl2::cfp(5ng); myo-3p::venus(10ng)]*	
JN5718	*sek-1(km4) X.; peEx5718[str-2p::sek-1::sl2::cfp(5ng); myo-3p::venus(10ng)]*	
JN5719	*sek-1(km4) X.; peEx5719[sre-1p::sek-1::sl2::cfp(5ng); myo-3p::venus(10ng)]*	
JN5720	*sek-1(km4) X.; peEx5720[ges-1p::sek-1::sl2::cfp(5ng); myo-3p::venus(10ng)]*	
JN5721	*sek-1(km4) X.; peEx5721[ins-1p::sek-1::sl2::cfp(5ng); myo-3p::venus(10ng)]*	
JN5722	*sek-1(km4) X.; peEx5722[daf-7p::sek-1::sl2::cfp(5ng); myo-3p::venus(10ng)]*	
JN5723	*sek-1(km4) X.; peEx5723[nhr-83p::sek-1::sl2::cfp(5ng); myo-3p::venus(10ng)]*	
JN5724	*sek-1(km4) X.; peEx5724[ges-1p::sek-1::sl2::cfp(5ng); rgef-1p::sek-1::sl2::cfp(5ng); myo-3p::venus(10ng)]*	
JN5725	*sek-1(km4) X.; peEx5725[ges-1p::sek-1::sl2::cfp(5ng); odr-3p::sek-1::sl2::cfp(5ng); myo-3p::venus(10ng)]*	
JN5726	*sek-1(km4) X.; peEx5726[glr-1p::sek-1::sl2::cfp(5ng); myo-3p::venus(10ng)]*	
JN5727	*grk-2(gk268) III.; peEx5727[odr-3p::grk-2::sl2::cfp(5ng); myo-3p::venus(10ng)]*	
JN5728	*grk-2(gk268) III.; peEx5728[sra-6p::grk-2::sl2::cfp(5ng); myo-3p::venus(10ng)]*	
JN5729	*grk-2(gk268) III.; peEx5729[gcy-5p::grk-2::sl2::cfp(5ng); myo-3p::venus(10ng)]*	
	Is[nlp-3 myo-3p::venus]	
	npr-15(tm2048); Is[nlp-3 myo-3p::venus]	
JN5730	*tir-1(tm3036) III.; sek-1(km4) X.*	Double Mutant Strain
JN5731	*nsy-1(ag3) II; sek-1(km4) X.*	
JN5732	*kgb-1(um3) IV.; sek-1(km4) X.*	
JN5733	*grk-2(gk268) III.; sek-1(km4) X.*	
JN5734	*mlk-1(ok2471) V.; sek-1(ag1) X.*	
JN5735	*kgb-1(um3) IV.; mlk-1(ok2471) V.*	
JN5736	*nlp-3(tm3023) X.; sek-1(km4) X.*	
JN5737	*npr-12 (tm1498) IV.; sek-1(km4) X.*	
JN5738	*npr-15 (tm2048) III. sek-1(km4) X.*	

Supplementary Table S2. Promoters used in rescue experiments

Promoter	Expression pattern
rgef-1	pan-neuron
ges-1	Intestine
odr-3	ASH, ADF, AWB
sra-6	ASH, PVQ
ocr-2	ASH, ADF, ADL
gcy-5	ASER
gcy-7	ASEL
gcy-8	AFD
str-1	AWB
str-2	AWC
sre-1	ADL
daf-7	ASI
ins-1	ASH, ASI, AIA
nhr-83	ASG
glr-1	PVQ

Supplementary Table S3. Primers used in Real-Time PCR

Oligo Name	Sequence
eef-1A.1 Fw:	ATTGCCACACCGCTCACA
eef-1A. 1 Rv:	CCGGTACGACGGTCAACCT
nlp-3. Fw	ATCGTCGCTTGCTTGGTCTT
nlp-3. Rv	TCAAGGAACGGGTTGACAGC
nlp-14. Fw	ACGGCCAAGGTTTCGGATTT
nlp-14. Rv	CTAGACCGTCAAGAGCTCGC
nlp-47. Fw	GTGCTCTGCTTCCTGGTTCT
nlp-47. Rv	CTGCTTGGACACGGTCTGAT

Supplementary Table S4. Immobility index of main strains

Strain	Immobility Index, Mean ± SEM (N = 6 assays)
N2 (wild type)	0.0810±0.0244
unc-43(e408)	0.8218±0.0573
tir-1(tm3036)	0.3214±0.0923
nsy-1(ag3)	0.3814±0.0401
sek-1(km4)	0.3679±0.0595
sek-1(km4);<br>*Ex [rgef-1p::sek-1]*	0.4323±0.0922
mek-1(pe3745)	0.2067±0.0677
mlk-1(ok2471)	0.1713±0.0378
kgb-1(um3)	0.5876±0.1990
grk-2(gk268)	0.0720±0.0332
nlp-3(tm3023)	0.0789±0.0769
npr-4 (tm1782)	0.1964±0.1411
npr-12 (tm1498)	0.3856±0.1428
npr-15 (tm2048)	0.1644±0.1186
