# Supplementary material for: Multiple p38/JNK mitogen-activated protein kinase (MAPK) signaling pathways mediate salt chemotaxis learning in C. elegans
Source: G3 (Bethesda). 2023 Jun 13;13(9):jkad129. doi: 10.1093/g3journal/jkad129 (PMC10468299; doi:10.1093/g3journal/jkad129)
Supplement: jkad129_Supplementary_Data [file jkad129_supplementary_data.zip › Supplemental_Material_Legends_G3-2023-404264.docx]

**Supporting information captions**

**Figure S1. Salt Chemotaxis Learning Assay**

Salt chemotaxis learning assay used in this study. (a) Adult worms were transferred to NGM plates with low (25 mM) and high (100 mM) salt concentrations in the absence or presence of food for 6 h, which is called conditioning. After conditioning, the worms were placed in the center of a 9 cm test plate (shown in b) and allowed to crawl for 45 min. (c) To prepare the test plate with a salt gradient, two cylindrical agar blocks with 0 mM NaCl or 150 mM NaCl were placed at points A and B. After 22 h at 20℃, a salt gradient was formed from 35 mM (point A) to 95 mM (point B).

**Figure S2. components of the JNK and p38 MAPK pathways**

All components of the JNK (left) and p38 MAPK pathways (right) identified in *C. elegans.*

Although DLK-1, the ortholog of mammalian MAP3K12, and MKK-4, the ortholog of mammalian MAP2K4, are involved in the p38 cascade in *C. elegans*, their ortholog in mammals is involved in the regulation of JNK signaling.

**Figure S3. Mutants of *jkk-1, dlk-1, and mkk-4* exhibit normal salt chemotaxis learning**

Mutants of *jkk-1* (a), a MAPKK in the JNK pathway, *dlk-1* and *mkk-4* (b), a MAPKKK and a MAPKK in the p38 MAPK pathway, do not exhibit defects in salt chemotaxis learning. The bars and error bars represent the mean values and SEM, respectively. ANOVA with Dunnett’s post hoc test (vs wild-type): N = 9 assays.

**Figure S4. Expression pattern of *sek-1::sl2::cfp* driven by the *odr-3* promoter.**

Although the *odr-3* promoter is known to drive expression mainly in AWC, in this study, the *odr-3* promoter abundantly drove expression of *sek-1::sl2::cfp* cDNA in ASH neurons (yellow arrow), slightly but detectably expressed in ADF and AWB neurons. However, no expression was detected in AWC. We suspect that expression sites driven by the *odr-3* promoter may depend on 3’UTR sequences inserted downstream of ORF. The *unc-54* 3’UTR was used in many studies for transgene expression, whereas we used *unc-2* 3’UTR for *sek-1::sl2::cfp* cDNA expression using the *odr-3* promoter.

(Cyan: CFP; Red: DiO)

**Figure S5. The site of action of SEK-1 in low-salt avoidance learning is different.**

Cell-specific rescue experiments of salt chemotaxis learning defects in the *sek-1* mutant. (a) The low-salt avoidance learning defect could be rescued only by *sek-1* expression using the *sra-6* promoter. Most transgenes with successful rescue of the high-salt attractive learning defect failed to rescue the low-salt avoidance defect. (b) Some promoters were used to mimic the expression pattern of the *sra-6* promoter; low-salt avoidance learning could not be significantly restored. The bars and error bars represent the mean values and SEM, respectively. ANOVA with Bonferroni’s post hoc test (Tg- vs Tg+). **P < 0.01. N = 9 assays.

**Figure S6. Comparison of *nlp-3* expression in the wild type and the *sek-1 (km4)* mutant before and after conditioning.**

A qRT-PCR analysis of *nlp-3*, *nlp-14*, and *nlp-47* mRNA levels in control and conditioned (100 mM / Fed) young adult wild-type and *sek-1(km4)* worms. The levels of these mRNAs are normalized to the levels of the *eef-1* mRNA. The bars and error bars represent the mean values and SEM, respectively. Kruskal*–*Wallis and Mann*–*Whitney tests. N = 12 assays.

**Figure S7. Model of interaction between sensory neurons involved in regulating salt chemotaxis learning by SEK-1 signaling and interneurons involved in salt chemotaxis learning.**

ASER sensory neuron sends synaptic input to AIA, AIB, and AIY interneurons; ASH sensory neurons send synaptic input to AIA, AIB, and AIZ interneurons; ADF sensory neurons send synaptic input to AIA, AIY, and AIZ interneurons. ASER, ASH, and ADF collectively connect with interneuron AIA.

**Figure S8. Salt chemotaxis after conditioning with 50 mM NaCl.**

Mutants of *sek-1*, which exhibit high salt chemotaxis defect after high-salt (100 mM) or low-salt (25 mM) learning, also show lower chemotaxis index after conditioning at 50 mM, a medium salt concentration. The bars and error bars represent the mean values and SEM, respectively. ANOVA with Dunnett’s post hoc test (vs wild-type). ***P < 0.001 N = 9 assays.

**Table S1. Strains and transgenic lines**

**Table S2. Promoters used in rescue experiments**

**Table S3. Primers used in Real-Time PCR**

**Table S4. Immobility index of main strains**
